# Extremely premature infants with patent ductus arteriosus closure: Comparative analysis of surgical ligation versus cardiac catheterization-based closure

**DOI:** 10.1097/MD.0000000000029103

**Published:** 2022-03-25

**Authors:** Rajesh Pandey, Lydia Youmans, Chiamaka Aneji, Cynthia Bell, John Breinholt, Amir Khan

**Affiliations:** a *McGovern School of Medicine, University of Texas, Health Science Center at Houston, Houston, TX,*; b *Phoenix Children's Hospital, Phoenix, AZ.*

**Keywords:** catheter based closure of patent ductus arteriosus, extremely low birth weight infant, extremely premature infants, hemodynamic decompensation after patent ductus arteriosus closure, patent ductus arteriosus, surgical ligation of patent ductus arteriosus

## Abstract

Our objective was to compare postprocedure hemodynamic decompensation in extremely premature infants who had their patent ductus arteriosus (PDA) closed with surgical ligation (SL) versus cardiac catheter-based closure (CCC).

This is a single-center retrospective review of extremely premature ( < 28 weeks) infants who had their PDA closed by SL or CCC.

Of the total of 69 infants, 53 underwent SL, and 16 had CCC. Infants in 2 groups were comparable at birth. However, at the time of the procedure, infants in the SL group were smaller, less mature, and had higher respiratory support. Vasopressor use, both pre- and postprocedure, was more common in the SL group. Nineteen percent of the infants in the SL group, compared to 6% in the CCC group (*P* = .34), required dose escalation or use of vasopressors after the PDA closure.

There was no significant difference between the 2 groups in postoperative hemodynamic decompensation. Large, multicenter, prospective study or randomized control trial will help to confirm our findings.

## 1. Introduction

There is a wide variation in patent ductus arteriosus (PDA) management in premature infants.^[1]^ PDA is reportedly associated with increased mortality and morbidities in preterm infants.^[2,3]^ However, treatment of PDA is not consistently associated with improved survival or disabilities.^[4-9]^ The role of medical and surgical management of PDA is often debated.^[3,10]^ Nevertheless, preterm infants continue to receive medical and surgical treatment of the PDA.

Surgical ligation (SL) of PDA is done for immediate and permanent closure of the ductus arteriosus. However, SL carries the risks of postoperative hemodynamic decompensation, vocal cord paralysis, and diaphragm injury. SL is also associated with neurodevelopmental impairments.^[10-12]^ Interest in minimizing those complications has led to an increase in CCC. CCC is a relatively new approach for managing PDA in extremely premature infants, and there are limited publications in this field.^[13-16]^ The primary aim of this study was to compare postprocedure hemodynamic decompensation in extremely premature infants who had their PDA closed with SL versus the CCC approach.

## 2. Methodology

We included extremely premature infants born between January 2012 and June 2017 who had their PDA closed by either SL or CCC. Historically, our institution performed SL for the closure of PDA. In 2014, we started CCC for PDA closure in extremely premature infants. In the CCC epoch, CCC was the preferred procedure for the closure of PDA. Still, a subset of infants underwent SL, especially if the infant was deemed unstable for transport to the cardiac catheterization suite. The diagnosis of PDA was based on clinical and echocardiographic findings. The decision to close the PDA and to use the vasopressors was at the discretion of attending neonatologists. In both epochs, only infants with symptomatic PDA underwent PDA closure. Generally, either surgical or catheter-based closure was done after failed medical treatment or medical treatment was contraindicated. Ibuprofen was the most commonly used medicine to treat PDA, followed by indomethacin. We excluded infants born after 28 weeks’ gestational age and infants with complex cardiac diseases. This study was approved by the institutional review board.

The primary outcome of the study was to compare postoperative hemodynamic decompensation between the SL (epoch: 2012-2014) and CCC (epoch: 2015-2017) groups. We defined postoperative hemodynamic decompensation as low blood pressure and decreased perfusion, rather than a number, which prompted dose escalation or vasopressor use (dopamine, epinephrine, dobutamine, or corticosteroids) after the procedure.

Our secondary outcomes were death before discharge, bronchopulmonary dysplasia, necrotizing enterocolitis, retinopathy of prematurity, intraventricular hemorrhage (IVH), severe IVH (defined as grade III or IV IVH with or without cystic changes) on head ultrasound.

We performed multiple exploratory analyses. Infants with PDA are treated at different postnatal ages and with different techniques (SL vs CCC). To evaluate the effects of timing of PDA closure, we compared infants with PDA closed at the following times:

- In the first 14 days of life (very early closure)
- Between 14 and 28 days of life (early closure)
- After 28 days of life (late closure)

Descriptive statistics were used to summarize the data as median (minimum, maximum) for continuous variables and count (percentage of total) for categorical variables. Differences between groups were tested using the Wilcoxon Rank Sum test for continuous data and chi-square or Fisher exact test for categorical data. We completed analyses in StataCorp. 2017. *Stata Statistical Software: Release 15*. College Station, TX: StataCorp LLC 15 SE, and statistical significance was defined as a two-tailed *P*-value < .05.

## 3. Results

Of 69 extremely premature infants included in the study, 53 had SL, and 16 had CCC for PDA closure. Infants in the 2 groups were comparable for birth weight (median 660 g vs 725 g), gestational age (25 weeks vs 25.4 weeks). However, at the time of the procedure, infants in the SL group were smaller (1240 vs 2045 g, *P* = < .001), less mature (postmenstrual age 31.9 vs 36.9 weeks, *P* = < .001), and were on higher respiratory support (85% intubated vs 44% in the CCC group). Infants in the SL group had larger PDA (median size 4 mm vs 3 mm, *P* = .01). Vasopressor use was more common in the SL group before (28% SL vs 6% CCC, *P* = .09) and after (40% SL vs 13% CCC, *P* = .06) the procedure (Table 1). Nineteen percent of infants in the SL group and 6% in the CCC group (*P* = .34) required an increased dose of vasopressors (Table 2).

**Table 1:** Baseline characteristics and clinical features.

		**Device closure (n = 16)**	**Surgery (n = 53)**	***P*-value**
Birth weight (g)*		725 (540, 1020)	660 (420, 1200)	.08
Gestational age (wk)*		25.4 (23.0, 27.3)	25.0 (22.9, 27.9)	.67
Multiple birth		8 (50%)	17 (32%)	.19
Inborn		11 (69%)	38 (72%)	.82
Male gender		8 (50%)	31 (58%)	.55
Antenatal steroids		12 (75%)	45 (85%)	.24
1 min APGAR		5 (1, 8)	3 (1, 9)	.57
5 min APGAR		7 (1, 9)	7 (3, 9)	.54
Postnatal steroids		9 (56%)	37 (70%)	.37
Severe IVH		3 (19%)	7 (13%)	.69
Sepsis		6 (38%)	18 (34%)	.77
SIP		1 (6%)	13 (25%)	.16
NEC		4 (25%)	8 (15%)	.45
Weight at time of procedure (g)		2045 (1120, 4970)	1240 (650, 4565)	< .001
Age at procedure (wk)*		11 (7, 23.3)	7.3 (2.7, 59.9)	< .001
PMA at procedure (wk)*		36.9 (32, 49.6)	31.9 (26.6, 87.4)	< .001
Preop PDA size (mm)*		4 (1, 5)	2.5 (1, 5)	.00
1		4 (25%)	1 (2%)	< .001
2		4 (25%)	2 (4%)
3		5 (31%)	5 (31%)
4		0	0
5		3 (19%)	3 (19%)
Previous PDA management		6 (38%)	25 (47%)	.57
	Room air	2 (13%)	0	.01
	Nasal cannula	2 (13%)	1 (2%)
	HFNC	3 (19%)	3 (6%)
	CPAP	2 (13%)	4 (8%)
	NIPPV or CMV	7 (44%)	43 (81%)
	HFOV	0	3 (4%)

CMV = conventional mechanical ventilation, CPAP = continuous positive airway pressure, HFNC = high flow nasal cannula, HFOV = high frequency oscillatory ventilation, IVH = intraventricular hemorrhage, NEC = necrotizing enterocolitis, NIPPV = noninvasive positive pressure ventilation, PDA = patent ductus arteriosus, PMA = postmenstrual age, SIP = spontaneous intestinal perforation.

^*^Expressed in median (range).

**Table 2:** Outcomes of infants.

		**Device closure (n = 16)**	**Surgery (n = 53)**	***P*-value**
Preprocedure inotropes use (including hydrocortisone)		1 (6%)	15 (28%)	.09
Preprocedure inotropes use (excluding hydrocortisone)		0 (0%)	7 (13%)	.18
Postprocedure inotropic use ( ≥ 72 h postintervention)		2 (13%)	21 (40%)	.06
Change in inotropic dose (including preprocedure hydrocortisone use)	
	More inotropes post	1 (6%)	10 (19%)	.34
	No change pre to post	14 (88%)	35 (66%)
	Less/no inotropes post	1 (6%)	8 (15%)
Change in inotropic dose (not including preprocedure hydrocortisone use)	
	More inotropes post	2 (13%)	16 (30%)	.20
	No change pre to post	14 (88%)	33 (62%)
	Less/no inotropes post	0 (0%)	4 (8%)
Postop intubation days		9 (1, 125)	11 (2, 370)	.18
Postop intubation > 72 h		11 (69%)	48 (91%)	.04
Length of stay (wk)		24.6 (7.6, 48.3)	22.7 (4.1, 84.0)	.58
CGA at time of discharge (wk)*		49.4 (32.1, 71.3)	46.1 (31.6, 111.6)	.51
BPD		16 (100%)	50 (94%) 1.00
ROP requiring treatment		2 (13%)	13 (24%)	.43
Death at hospital		2 (13%)	4 (8%)	.61

BPD = bronchopulmonary dysplasia, CGA = corrected gestational age, ROP = retinopathy of prematurity.

^*^Includes discharge, transfer, or death.

When analyzed based on the timing of PDA closure, none of the infants in our study had their PDA closed in the first 14 days of life. Eight infants (12%) had their PDA closed early, between 14 and 28 days of life, all by SL. Sixty-one of 69 (88%) patients had their ductus closed late, after 28 days of life. In the late repair group, 16 infants had CCC, and 45 had SL. To evaluate outcomes based on the procedure type, we compared infants in catheter based closure (n = 16) group with infants in the SL (n = 45) group, who had their PDA closed after 28 days (Table 3). Both groups had comparable birth weight and gestational age at birth. However, at the time of the procedure, infants in the SL group were smaller, less mature, and had higher respiratory support. There were no differences in primary and secondary outcomes (Table 4).

**Table 3:** Baseline characteristics and clinical features of the late repair subgroup (PDA closed after 28d) device vs surgical ligation.

		**Device closure (n = 16)**	**Surgery (n = 45)**	***P*-value**
Birth weight (g)*		725 (540, 1020)	670 (420, 1120)	.07
Gestational age (wk)*		25.4 (23, 27.3)	25.0 (22.9, 27.9)	.69
Male gender		8 (50%)	25 (56%)	.70
Antenatal steroids		12 (75%)	39 (87%)	.28
5 min APGAR		7 (3, 9)	7 (1, 9)	.60
Postnatal steroids		9 (56%)	34 (76%)	.15
Weight at time of procedure (g)*		2045 (1120, 4970)	1250 (710, 4565)	< .001
Age at procedure (wk)*		11 (7, 23.3)	7.6 (4.1, 59.9)	< .001
CGA at procedure (wk)*		36.9, 32, 49.6)	32.6 (29.3, 87.4)	< .001
Preop PDA size*(mm)		2.5 (1, 5)	4 (1, 5)	.001
1		4 (25%)	1 (2%)	.001
2		4 (25%)	2 (4%)
3		5 (31%)	12 (27%)
4		0 (0%)	14 (31%)
5		3 (19%)	16 (36%)
Previous PDA management		6 (38%)	20 (44%)	.63
Preprocedure respiratory support				
	Room air	2 (13%)	0 (0%)	.02
	Nasal cannula	2 (13%)	1 (2%)
	HFNC	3 (19%)	3 (7%)
	CPAP	2 (13%)	4 (9%)
	NIPPV/CMV	7 (44%)	35 (78%)
	HFOV	0 (0%)	2 (4%)

CGA = corrected gestational age, CMV = conventional mechanical ventilation, CPAP = continuous positive airway pressure, HFNC = high flow nasal cannula, HFOV = high frequency oscillatory ventilation, NIPPV = non invasive positive pressure ventilation, PDA = patent ductus arteriosus.

^*^Expressed in median (range).

**Table 4:** Outcomes of the late repair subgroup (PDA closed after 28d) device vs surgical ligation.

		**Device closure (n = 16)**	**Surgery (n = 45)**	***P*-value**
Preprocedure inotropes use (including hydrocortisone)		1 (6%)	10 (22%)	.26
Preprocedure inotropes use (excluding hydrocortisone)		0 (0%)	2 (4%)	1.00
Postprocedure inotropic use ( ≥ 72 h postintervention)		2 (13%)	16 (36%)	.11
Change in inotropic dose (including preprocedure hydrocortisone use)				
	More inotropes post	1 (6%)	9 (20%)	.48
	No change pre to post	14 (88%)	32 (71%)
	Less/no inotropes post	1 (6%)	4 (9%)
Change in inotropic dose (not including preprocedure hydrocortisone use)				
	More inotropes post	2 (13%)	15 (33%)	.11
	No change pre to post	14 (88%)	30 (67%)
	Less/no inotropes post	0 (0%)	0 (0%)
Postop intubation days		9 (1, 125)	11 (2, 370)	.16
Postop intubation > 72 h		11 (69%)	40 (89%)	.06
Length of stay (wk)		24.6 (7.6, 48.3)	24 (12.9, 84)	.92
CGA at time of discharge (wk)*		49.4 (32.1, 71.3)	48.3 (38.1, 111.6)	.83
BPD		16 (100%)	43 (96%)	1.00
ROP requiring treatment		2 (13%)	12 (27%)	.33
Death at hospital		2 (13%)	4 (9%)	.65

BPD = bronchopulmonary dysplasia, CGA = corrected gestational age, PDA = patent ductus arteriosus, ROP = retinopathy of prematurity.

^*^Includes discharge, transfer, or death.

The early SL (n = 8) group was comparable to the late SL (n = 45) group for birth weight, gestational age. At the time of the procedure, infants who had their PDA ligated early were smaller (850 vs 1250 g, *P* = .006) and more likely to be on vasopressors (Table 5). The early SL group was less likely to require dose escalation of vasopressors than the late SL group. The early SL group had a shorter length of stay and were discharged about 8 weeks before the late SL group (40.5 vs 48.3 weeks’ postmenstrual age, *P* = .02) (Table 5).

**Table 5:** Baseline characteristics, clinical features, and outcomes of the early vs late repair subgroup (PDA closure 14-28d vs more than 28d).

		**Early repair (n = 8)**	**Late repair (n = 45)**	***P*-value**
Birth weight (g)*		610 (548, 1200)	670 (420, 1120)	.82
Gestational age (wk)		24.9 (23.6, 27.4)	25 (22.9, 27.9)	.99
Weight at time of procedure (g)		850 (650, 1390)	1250 (710, 4565)	.01
Age at procedure (wk)*		3.2 (2.7, 4)	7.6 (4.1, 59.9)	< .001
Preop PDA size (mm)*		3 (3, 4)	4 (1, 5)	.03
Previous PDA management		6 (63%)	20 (44%)	.45
Preprocedure respiratory support			
	NC/HFNC	0 (0%)	4 (9%)	1.00
	CPAP	0 (0%)	4 (9%)
	Intubated/NIPPV	8 (100%)	35 (78%)
	HFOV	0 (0%)	2 (4%)
Outcome			
Preprocedure inotropes use (including hydrocortisone)		5 (63%)	10 (22%)	.03
Preprocedure inotropes use (excluding hydrocortisone)		5 (63%)	2 (4%)	< .001
Postprocedure inotropic use ( ≥ 72 h postintervention)		5 (63%)	16 (36%)	.24
Change in inotropic dose (including preprocedure hydrocortisone use)			
	More inotropes post	1 (13%)	9 (20%)	.03
	No change pre to post	3 (38%)	32 (71%)
	Less/no inotropes post	4 (50%)	4 (9%)
Change in inotropic dose (not including preprocedure hydrocortisone use)			
	More inotropes post	1 (13%)	15 (33%)	< .001
	No change pre to post	3 (38%)	30 (67%)
	Less/no inotropes post	4 (50%)	0 (0%)
Postop intubation days		12.5 (4, 39)	11 (2, 370)	.54
Postop intubation > 72 h		8 (100%)	40 (89%)	1.00
PMA at time of discharge (wk)†		40.5 (31.6, 55.3)	48.3 (38.1, 111.6)	.02
BPD		7 (88%)	43 (96%)	.16
Death at hospital		0 (0%)	4 (9%)	1.00

BPD = bronchopulmonary dysplasia, CPAP = continuous positive airway pressure, HFNC = high flow nasal cannula, HFOV = high frequency oscillatory ventilation, NC = Nasal Canula, NIPPV = non invasive positive pressure ventilation, PDA = patent ductus arteriosus.

^*^Expressed in median (range).

^†^Includes discharge, transfer, or death.

We performed an exploratory analysis to estimate the effects of hydrocortisone use before the procedure and the incidence of postoperative hemodynamic decompensation. Generally, preoperative hydrocortisone was prescribed if the dose of dopamine infusion was > 10μmg/kg/min or the infant was receiving hydrocortisone for underlying adrenal insufficiently/chronic lung disease. Fewer infants who were on hydrocortisone before PDA closure developed hemodynamic decompensation (16% vs 26%) than those who were not (Table 2). A similar pattern was seen in the SL group. Nineteen percent of the infants who were on hydrocortisone before the PDA closure developed hemodynamic decompensation compared to 30% of infants who were not (Table 5).

## 4. Discussions

Procedural details and immediate perioperative outcomes from our center have been previously published.^[17]^ Infants in the SL group were smaller and less mature at the time of the procedure. They had larger PDA and were on higher respiratory support at the time of PDA closure than the infants in the catheter-based closure group. We did not find a difference in postoperative hemodynamic decompensation between the CCC group and the SL group (30% vs 13%, *P* = .2). Our finding is different from a recent report where authors reported the need for higher postoperative hemodynamic support on the SL group than the catheter-based closure group of very low birth weight infants.^[18]^ Fewer infants in the catheter-based closure groups remained intubated for more than 72hours (69% vs 91%, *P* = .04). Our findings are similar to a recent retrospective review where the authors reported improved respiratory outcomes in catheterbased device closure of PDA.^[19]^

In our study, infants in the SL groups were smaller and less mature, at the time of the procedure, than the infants in the catheter-based closure group, but the 2 groups were comparable at birth. This finding is similar to what has been reported elsewhere.^[20]^ This can be explained by the fact that catheterbased closure is not often performed in the tiniest patients in part due to concerns for instability during transport to the catheterization suite and size-related technical difficulties. In contrast, SL can be done on a critically ill, tiny infant at the bedside.

When we compared the early versus late SL group outcomes, the early surgical-ligation group had a shorter length of stay (16 weeks vs 24weeks) than the late surgical-ligation group. This finding is statistically significant and clinically meaningful. Our results are comparable to conclusions from Saldeno et al.^[21]^ In a retrospective review of extremely premature infants, the authors compared infants with prolonged, persistent PDA (defined as open PDA for at least 21 days of life) with matched infants. The latter had their PDA closed by 21 days of life, spontaneously or medically, or surgically. The mean gestational age at birth was 25 weeks, and the birth weight was 860 g. The authors reported that infants with prolonged PDA had a longer hospital stay (142 ±62 vs 85±15 days, *P* = .012). It is not easy to determine whether the delayed closure of PDA or biological factors of the patients that lead to delayed closure of PDA contributed to the difference in length of stay. Nonetheless, a significantly shorter duration of hospitalization associated with early closure of PDA warrants further studies.

We found that infants who were on hydrocortisone before the procedure were less likely to develop hemodynamic decompensation after the procedure compared to infants who were not on hydrocortisone. Our findings are similar to reported results.^[22]^

Our study has several limitations. We acknowledge the potential for bias due to the retrospective nature of this study, but as either SL or catheter-based closure was done in 1 epoch, there was no referral bias from our institution. The decision to refer for surgery or catheter-based closure group from outside hospitals depended on the attending neonatologist's preference, and there were no standard criteria. The decisions to treat PDA and use vasopressors were not standardized and were based on the discretion of attending neonatologists. Our small sample size might have limited the ability to detect actual differences between the groups. We could not adjust the outcomes for differences in baseline characteristics due to a small number of subjects. The results of catheter-based closure depend on the expertise and experience of the interventional cardiologist, and our findings may not be replicable.

## 5. Conclusion

We did not find statistically significant and clinically meaningful differences between the SL and the catheter-based closure groups. Among infants with SL, infants who have their PDA closed between 14 and 28 days of life had significantly shorter hospital days than infants who had their PDA closed after 28 days of life. Preoperative use of hydrocortisone may decrease postoperative hemodynamic decompensation. Further studies are warranted to answer these questions. Adequately powered randomized control trial is needed to confirm our findings.

## Author contributions

**Conceptualization:** Rajesh Pandey, Chiamaka Aneji, John Breinholt, Amir Khan.

**Data curation:** Lydia Youmans.

**Formal analysis:** Cynthia Bell.

**Investigation:** Rajesh Pandey, Chiamaka Aneji, Cynthia Bell.

**Methodology:** Rajesh Pandey, Lydia Youmans, Cynthia Bell, John Breinholt.

**Validation:** Lydia Youmans.

**Writing** - **review & editing:** Lydia Youmans, Chiamaka Aneji, John Breinholt, Amir Khan.
